# Past, Present, and Future of Mechanical Dentistry

**Published:** 1905-10

**Authors:** 


### Editorial.

### PAST, PRESENT, AND FUTURE OF MECHANICAL DENTISTRY.

The question of serious import in dentistry at the present time is, What shall constitute the fundamentals in teaching and practice? The dental world is apparently fast drifting into three classes. One regards mechanical dentistry, in its broadest sense, including operative, as the foundation of all our work and the one thing most necessary in the equipment of every practitioner. Another holds that this is altogether a minor qualification,—in fact, may be entirely set aside as unworthy and left exclusively to the one with mechanical tastes and experience. This man regards the medical school and all that is therein implied as the foundation of dentistry, and that nothing more is needed. The third holds that dentistry is a complex profession based on mechanics, nurtured through many years upon the idea that teeth to be saved must be defended from the inroads of destructive environments through the mechanical substitution of lost parts, and, failing in this, after an indefinite period, through the same mechanical skill substituting for the lost teeth artificial dentures.

That medical science, in its various branches, is of vast importance in the building up of dentistry needs no argument with the advanced thinker of the present period, but that it covers all that is necessary in the training of the dentist cannot be conceded.

The dental practitioners of a half-century in the past had no difficulty with these several divisions in thought and practice. That this has been materially changed is unfortunate, as it has created an antipathy detrimental to the best progress of the art. At this period there was but one dentistry, and its bounds were well defined and thoroughly understood. In time there came into it men from the medical schools too late in life, as a rule, to become proficient in the mechanical and operative side of the profession. These, very naturally, sought to separate the purely medical and surgical from the mechanical and relegate the latter to the secondary position it properly occupied, in their estimation. This was strengthened by the views held in Continental Europe and also by the prejudice that has always existed against it by the medical profession universally and an unwillingness to recognize it as a part of the great healing art. It was assumed by this class that all connection with mechanical dentistry must be severed before it could be classed as a learned profession. While at present it has not gone so far as to include operations upon the natural organs, the evident tendency is eventually to place that in the same category with the worker upon the laboratory bench.

It seems to the writer that the time has arrived for a clear and positive affirmation, on the part of the constituted professional bodies, what should or should not be classified as dentistry. If the men who repudiate all mechanical knowledge are worthy to fill the honored place of practitioners of the dental art, it will be to the interest of the world to know it; but if, on the other hand, the said world cares very little for the scientific attainments of the individual, but does care very much for his ability in his mechanical operations, it will be of vast importance that this should be most thoroughly cultivated, that humanity may reap the benefit.

The writer occupies the position that there need be no real lines of demarcation between the purely mechanical, operative, scientific, and medical branches. They should be so completely dovetailed, each to the other, that the lines of separation should be indistinguishable. This is, beyond all question, the true stand to take. Neither of the four divisions can be lopped off without serious loss; indeed, each is indispensable to the perfection of the whole. It seems, therefore, that those who are working to effect a separation are straining to produce something based on false ideals, and these latter never can result satisfactorily.

Mechanical dentistry, in its broadest acceptation, has been one of the most important factors in the hygienic improvement of human life. Until mechanical dentistry began to assume proportions of value to the masses, their condition was of the most serious character. From the dawn of civilization the world had had no means of preserving, much less to have inserted a substitute for, lost organs. It is within the memory of many still living that there was a period when there existed a strong prejudice against any attempt to thwart the destructive action of what were called the natural processes of destruction, and that the word *false*, in connection with the human body, was regarded as antagonizing the will of the Creator, and he or she who attempted to subvert this will by any substitute came under suspicion and reproach. The early practitioners had, therefore, a difficult task, and one not appreciated at the present period of more intelligent enlightenment. The advantages of a substitute for lost dentures were but slowly comprehended. This was due, in a measure, to imperfect skill on the part of dental mechanicians, for ability in this direction was of slow growth. As this was developed through years of experimentation, there came a different feeling, prejudices slowly vanished, and the skill of the mechanical dentist was more and more brought into requisition.

Upon the advent of the rubber base for dentures there came an increased, but in some respects an inferior, mechanical ability. The skill of the jeweller, the pride of the earlier dentist, was lost and has never yet been wholly regained; in fact, it is mainly due to this that the demand has been made for a separation from mechanics. While it is true much has been lost through the advent of rubber for dental purposes, much has been gained. That which has been lost to dentistry has been to the increase of health and happiness of large numbers of the human family. Dental workers have been multiplied by the thousands, enabling other thousands to have artificial substitutes at very moderate cost, not possible fifty years ago.

The result of all this work is beginning to make itself manifest in a remarkable manner. The individual who has passed the years of the Psalmist can well recall the appearance of the grandfathers and grandmothers of his youth. Bowed with the infirmities of age at sixty, toothless and with elongated mandibles, these caricatures of humanity were doomed to give their comparatively short lives as an evidence of the fact that nature is far from perfect, and that man, in numerous instances, is required to step in and remedy destructive effects. It was everywhere in evidence that the old men and women, through lack of proper digestive ability, failed rapidly physically and mentally. The so-called period of dotage was expected as a matter of course. The individual of middle life looked forward to this time with a feeling of horror. To the woman it was the period of the mob-cap and the witch's face. This the mechanical dentist has changed to a large degree, and eventually must be a condition obliterated. All that he has done has not been artistic,—far from it,—but he has given the larger number the means of masticating food properly. He has enabled the wearer of an artificial denture to be assured that, however imperfect it might be, it will aid, better than any other means, in keeping the stomach in reasonably good order, and with this in proper condition, mental force may be indefinitely continued. It is recognized that there are other factors that enter into and produce a loss of mental vigor, but it is nevertheless beyond question that, given a good digestion, mental activity will be prolonged to an age not possible, as a rule, a hundred years in the past.

It is then of vital importance that mechanical dentistry should not only be fostered to the fullest extent, but it should be held to be worthy the best efforts of the most cultivated. It is the art of all arts that appeals most to humanity, or should appeal, for it places the vestibule of the entire organism in a fit condition for the reception and comminution of the food necessary for the maintenance of the body in health. The same may be said equally of the operative branch; in fact, these two constitute a dentistry worthy the most extended efforts for their perfection.

Dentistry, as a whole, must be regarded as a failure if its mission is confined to the salvation of the natural teeth. While prophylaxis has, undoubtedly, a great future, and will be eventually a specialty in itself, it will fail, as all efforts have failed, to keep the natural organs to an advanced old age. Dentists, many of them at least, have yet to learn that the destructive processes are sure, in the future life of the individual, to end the life of the teeth. The exceptions to this are too few to be considered. Some operators still persist in regarding the destruction manifest after sixty as pyorrhœa alveolaris, but this is only in part true. The pulps die; the tubulated structure has long since ceased to fulfil its function, being closed and obliterated by secondary deposits; the teeth are no longer nourished by the peridental membrane; osteomalacia sets in, and the teeth become foreign bodies, are acted upon by the environments, and go down to destruction. The labor of the dentist has been of immense value in prolonging the usefulness of the teeth, but in the end it is written, failure.

The work of the mechanical dentist, then, is to fill this gap and make the skill of the dentist continuous. It commenced with the cradle, and it should follow humanity, through the added decades of life, to the grave.

With this view of prosthetic dentistry, and its twin brother operative dentistry, it seems almost a crime against humanity that there should be any effort to belittle this work. It should be welcomed as the handmaiden of our profession. And while dentistry, as a whole, may change, it seems reasonable to expect that the future of mechanical dentistry may be regarded as being coexistent with the life of the race on this planet. Therefore, while leaving nothing in the realm of scientific medicine untouched and unexplored, let us still cling to the foundation of that dentistry that has divested life of one of its many terrors and made the old man and the old woman more worthy of our respect and veneration, for the reason that our profession has made youth perennial.

